# Dietary mannan oligosaccharides strengthens intestinal immune barrier function *via* multipath cooperation during *Aeromonas Hydrophila* infection in grass carp (*Ctenopharyngodon Idella*)

**DOI:** 10.3389/fimmu.2022.1010221

**Published:** 2022-09-13

**Authors:** Zhi-Yuan Lu, Lin Feng, Wei-Dan Jiang, Pei Wu, Yang Liu, Jun Jiang, Sheng-Yao Kuang, Ling Tang, Shu-Wei Li, Cheng-Bo Zhong, Xiao-Qiu Zhou

**Affiliations:** ^1^ Animal Nutrition Institute, Sichuan Agricultural University, Chengdu, China; ^2^ Fish Nutrition and Safety Production University Key Laboratory of Sichuan Province, Sichuan Agricultural University, Chengdu, China; ^3^ Key Laboratory of Animal Disease-Resistance Nutrition, Ministry of Education, Ministry of Agriculture and Rural Affairs, Key Laboratory of Sichuan Province, Sichuan, China; ^4^ Sichuan Animal Science Academy, Sichuan Animtech Feed Co. Ltd, Chengdu, China; ^5^ Animal Breeding and Genetics Key Laboratory of Sichuan Province, Animal Nutrition Institute, Sichuan Academy of Animal Science, Chengdu, China

**Keywords:** mannan oligosaccharides, immune barrier, NFκB, TOR, Grass carp (*Ctenopharyngodon idella*)

## Abstract

In recent years, mannose oligosaccharide (MOS) as a functional additive is widely used in aquaculture, to enhance fish immunity. An evaluation of the effect of dietary MOS supplementation on the immune barrier function and related signaling molecules mechanism of grass carp (*Ctenopharyngodon idella*) was undertaken in the present study. Six diets with graded amounts of MOS supplementation (0, 200, 400, 600, 800, and 1000 mg/kg) were fed to 540 grass carp over 60 days. To examine the immune response and potential mechanisms of MOS supplementation on the intestine, a challenge test was conducted using injections of *Aeromonas hydrophila* for 14 days. Results of the study on the optimal supplementation with MOS were found as follows (1) MOS enhances immunity partly related to increasing antibacterial substances content and antimicrobial peptides expression; (2) MOS attenuates inflammatory response partly related to regulating the dynamic balance of intestinal inflammatory cytokines; (3) MOS regulates immune barrier function may partly be related to modulating TLRs/MyD88/NFκB and TOR/S6K1/4EBP signalling pathways. Finally, the current study concluded that MOS supplementation could improve fish intestinal immune barrier function under *Aeromonas hydrophila* infected conditions.

## Introduction

A growing global fish demand has prompted intensive aquaculture research. However, the intensive expansion of aquaculture is often accompanied by fish health/disease issues. Fish enteritis is one of the common diseases in intensive aquaculture, which leads to high mortality in farmed fish and causes huge annual economic losses in the world (1). Mannan oligosaccharides (MOS) is functional oligosaccharides, which has received considerable attention in recent years for its promotion of fish health (2). According to existing studies, we know that MOS has a beneficial effect on the immune function of multiple functional organs (such as head kidney, spleen, skin, etc.) in fish (3–6). Different from other functional organs, the intestinal tract, as a bridge connecting the internal and external environment of the body, can directly contact with foreign substances (7, 8). Hence, fish intestine is very susceptible to direct effects of feed composition. Studies have shown that mannose oligosaccharides contain mannose residues that can specifically bind to intestinal epithelial pattern recognition receptors (PRR) to induce immune responses (8, 9). Therefore, the appropriate level of MOS supplementation maybe involves the interaction of the intestinal epithelium to maintain intestinal health. Generally, intestinal health is closely related to the intestinal immune barrier. Several fragmentary reports have been published about the influence of MOS addition on the teleost intestinal immune barrier. According to these limited studies, in the intestine, MOS addition could up-regulate the immunoglobulin (Ig) and tumor necrosis factor (TNF)-α expression in European sea bass (*Dicentrarchus labrax*) (10), IL-10 in rockfish (*Sebastes schlegelii*) (11), MUC-2 in tropical gar (*Atractosteus tropicus*), TGF-β in turbot *Scophthalmus maximus* (12) and down-regulate IL-1β, IL-6 and IL-8 in European sea bass (13, 14). Although many intestinal inflammatory cytokines have been affected by MOS supplementation, the specific mode of action and related mechanisms in the intestine underlying the beneficial effects of MOS remains unclear in fish. Thus, a more comprehensive and in-depth exploration of the molecular mechanisms will be necessary to understand this relationship.

As is well-known, the intestinal immune barrier is related to its immune components such as antimicrobial compounds (e.g., acid phosphatase (ACP), lysozyme (LZ), complement 3 (C3) and C4), antimicrobial peptides (e.g., hepcidin, liver-expressed antimicrobial peptide (LEAP)-2A, and β-defensin), Ig production and T lymphocytes (4, 15). Apart from study looking into Ig, no evidence has been found that MOS supplementation affects the innate immune components of fish. A study on growing pig intestines indicated that MOS supplementation could increase Zn retention (16). A previous study from our laboratory confirmed that Zn could increase the content of IgM in the intestine of grass carp (17). It was also reported that MOS supplementation could increase phosphorus digestibility in the ileum of piglets (18). We have demonstrated in previous studies that phosphorus regulates hepcidin and LEAP2B mRNA levels in the head kidney and spleen of grass carps (19). To further build on these findings, further investigations into non-cellular immune components are needed to determine whether MOS influences fish immune barrier function in the intestinal tract.

In addition, the fish immune barrier is also strongly linked to intestinal cells (e.g., epithelial cells, mesoderm cells and immune cells) and multiple cytokines (19). Studies on human PBMCs indicated that cytokines are regulated by mammalian target of rapamycin (mTOR) signaling (20) and nuclear factor-κB (NFκB) signaling (21). There is, however, little information available regarding the effect of MOS supplementation on intestinal cytokines and the underlying mechanisms (NFκB and TOR pathway). It has been reported that the addition of MOS to turkey intestines increased butyrate concentrations (22). Butyrate has been found to down-regulate the mRNA levels of TNFα, IL-15, and the signaling molecule NFκBp65 in our lab in a prior study (23). The presence of prostaglandins is increased in the intestine of European sea bass with MOS addition (24). Another study showed that prostaglandin could increase mTOR protein levels in mouse hepatocytes (25). Furthermore, the Toll-like receptors (TLRs) family of proteins has emerged as one of the most important determinants of immune activation in immune response research (26). As of now, only one study has been published about the influence of MOS on fish intestinal TLR family proteins. The study showed that the gene expression of TLR3 was up-regulated with MOS supplementation in the intestine of juvenile hybrid grouper (*Epinephelus lanceolatus* ♂ × *Epinephelus fuscoguttatus* ♀) (27). Based on these data, a relationship may exist between dietary MOS supplementation and the inflammatory cytokines and related signalling molecules in fish, which requires further investigation.

Based on our previous work on the intestinal tract health and growth performance of grass carp with MOS supplementation (6, 28), the present study examined the effects of dietary MOS supplementation on intestinal immune barrier function in grass carp under *Aeromonas hydrophila* (*A. hydrophila*) challenged conditions. Specifically, we explored the impact of MOS addition on antimicrobial substances, multiple cytokines, and possible pathways in the fish intestine, which might shed light on the underlying mechanisms in the intestines as well as the effect of MOS on the immune response. Currently, grass carp ranks as one of the most popular aquaculture species (29). According to the FAO, grass carp production reached 5.7 million tons in 2018 (29). Consequently, this present study not only provides a scientific reference for grass carp commercial feed formulations, which is of great importance for grass carp cultivation but also offers a reliable theoretical foundation for the development of an oral vaccine.

## Materials and methods

### Experimental design

Preparation and storage methods of the MOS (Sciphar Hi-Tech Industry, Xi’an, purity: 99.12%) diet are based on our previous research (28). As shown in **Supplementary Table 1**, the formulation of the experimental diet and proximate composition analyses were conducted. In place of cornstarch, different levels of MOS (0, 200, 400, 600, 800, and 1000 mg/kg) were added to the basal diet. A completed diet was stored at 4°C until it was given as feed.

### Animals and experimental management

The procedures used in this research were allowed by the Animal Care Advisory Committee of Sichuan Agricultural University under permit No. LZY-2018114005 throughout the feeding trial. Tong Wei fisheries (Sichuan, China) provided the grass carp, which were adapted to fishpond culture conditions for a month before the experiment. 540 fish are randomly assigned to eighteen nylon cages [1.4 L × 1.4 W × 1.4 H (m)], resulting in 30 fish per cage, with the same feeding frequency and experimental period as previously described (28). The typical management parameters for a test are that the level of dissolved oxygen > 6.0 mg/L, the temperature of the water is 28.55°C ± 2.0°C, the pH value is 7.5 ± 0.3, and the experiment is conducted with a natural light cycle.

### Challenge trial

In accordance with our published work (6, 28), we conducted a 14-day challenge trial to assess the effects of dietary MOS on intestinal immune function after the growth trial. In brief, 1.0 ml of *A. hydrophila* (FDL20120711) was intraperitoneally injected into five randomly selected fish per replicate from each MOS group. Other conditions of the challenge trial were same as that of the feeding trial. In previous studies, we have successfully established *A. hydrophila* challenged models (28).

### Sample preparation and biochemical analysis

At the end of the challenge trial, all grass carp were anesthetized in a benzocaine bath. Then, the abdominal cavity was carefully opened and the intestine was removed immediately. After quickly removing the intestines of each fish, the proximal intestine (PI), middle intestine (MI) and distal intestine (DI) were separated, then fish intestine segments were rapidly collected and temporarily stored in liquid nitrogen. To facilitate later biochemical analysis, the samples were stored at -80°C. An intestine tissue homogenate containing 10% (w/v) saline (4°C) was centrifuged (6000 g, 20 minutes) to determine immune-related parameters. After that, the supernatant was collected. ACP, C3, C4, LZ, and IgM biochemical analysis methods are shown in **Supplementary Table 2**.

### Real-time PCR

The *q*RT-PCR was conducted in reference to our previous work (28). In brief, total RNA was isolated from intestinal segments by using Takara’s RNAisoPlus Kit (Dalian, China). The quality of the RNA was determined by electrophoresis on 1% agarose gels and then quantified by spectrophotometry at 260/280 nm using a Nanodrop 2000 (Thermo Scientific, USA). After RNA extraction, the PrimeScript**™** RT reagent kit was used (Takara, Dalian, China) to reverse-transcribe it into cDNA. **Supplementary Table 3** shows the primers we designed for *q*RT-PCR based on the sequences we previously cloned. As described previously, we screened four internal reference genes and selected β-Actin and GAPDH as our final two candidates (28). Calculation of target gene amplification efficiency was done according to manufacturer instructions after preparing melting curves. The transcription level of genes was calculated according to Livak and Schmittgen’s method (2^−ΔΔCT^) (30).

### Immunohistochemistry

To prepare the intestinal samples for immunohistochemical staining, fresh intestinal samples were fixed in 4% paraformaldehyde solution. Intestinal samples were prepared by Lilai Biotechnology (Sichuan, China) as paraffin sections. We purchased an IHC kit from BOSTER Biological Technology (SA1028, Wuhan, China). The process was as follows: in short, paraffin sections were dewaxed with xylene and rehydrated in a graded ethanol series before removing endogenous peroxidase activity with hydrogen peroxide 3%. Then, a microwave-repaired antigen was then incubated with EDTA-repair solution, followed by 5% BSA blocking solution, before being incubated overnight with a primary antibody. Following SABC incubation and biotin labeling, slices were created for DAB coloring and hematoxylin re-staining. As a final step, dehydration of the sections was accomplished with graded ethanol solutions, followed by xylene transparency, and finally sealing with neutral gum. A light microscope (TS100, Nikon, Tokyo, Japan) was used to acquire and visualize images of the prepared IHC slices after drying overnight at 60°C. The expression of p-IRAK1, MyD88, TRAF6, and NFκBp65 was quantified using Image Pro Plus 6.0 (Media Cybernetics, Inc., Rockville, MD, USA). For IHC measuring, each antibody is described in **Supplementary Table 4** is described in detail. The reagent instructions were followed and adjustments were made as necessary.

### Western blot analysis

Laboratory analysis and the operational parameters of the blotting of intestinal homogenates and antibodies were performed as previously described (4, 5). Extraction and determination of tissue proteins was done using the RIPA and BCA assay kit (Beyotime). The prepared samples (40 μg/lane) were transferred to a PVDF membrane after separation by 10% SDS-PAGE. The membranes were then incubated with primary antibodies overnight (4°C, 14h). Following membrane washing, incubation with secondary antibodies was done incubated at room temperature for 90 minutes. Based on the previous description, protein signals were then visualized and quantified using NIH Image J, 1.42q (4, 5, 28). The detailed description of all antibodies is listed in **Supplementary Table 5** of the current study.

### Statistical analysis

By using Levene’s test for homogeneity and the Shapiro-Wilk test for normal distribution, we assessed data homogeneity and normality. One-way ANOVA with Tukey’s multiple comparisons test was used to analyze biochemical, gene expression, and protein level data, while the Student t-test was used to analyze immunohistochemical data (IOD), *P* < 0.05 was considered significant. As previously described, data analyses were performed using PROC MIXED of SAS software version 9.4 (SAS Institute Inc. 2004) (28). The linear and quadratic effects of MOS supplementation were assessed using orthogonal polynomial contrasts. The visualization of antibiotic substance, cytokines, immunohistochemical, and correlation data was done with *R* Studio v4.0.2 and Hiplot (https://hiplot.com.com) (5).

## Result

### Growth and disease resistance phenotypes

In this study, we used the same growth trials as we used in our previous grass carp research (28). The effectiveness of MOS supplementation on fish growth performance was found to be a quadratic effect (*P* < 0.01) after the growth experimental period. In comparison with the control group, final body weight (21.59%), specific growth rate (16.24%) and percent weight gain (31.34%) of the optimum group (400 mg/kg MOS) were significantly elevated along with increased intestine length (14.82%), intestine weight (26.98%), intestine length index (10.41%) and intestinal somatic index (13.92%). All fish survived in all groups after exposure to *A. hydrophila* and enteritis morbidity showed a quadratic relationship with MOS addition (*P* < 0.05). It was found that the optimal group (400 mg/kg MOS) experienced a 53.45% decrease in enteritis morbidity, as well as a 42.56% decrease in red-skin morbidity (5, 28).

### Intestinal immunological parameters

**Figure 1:**
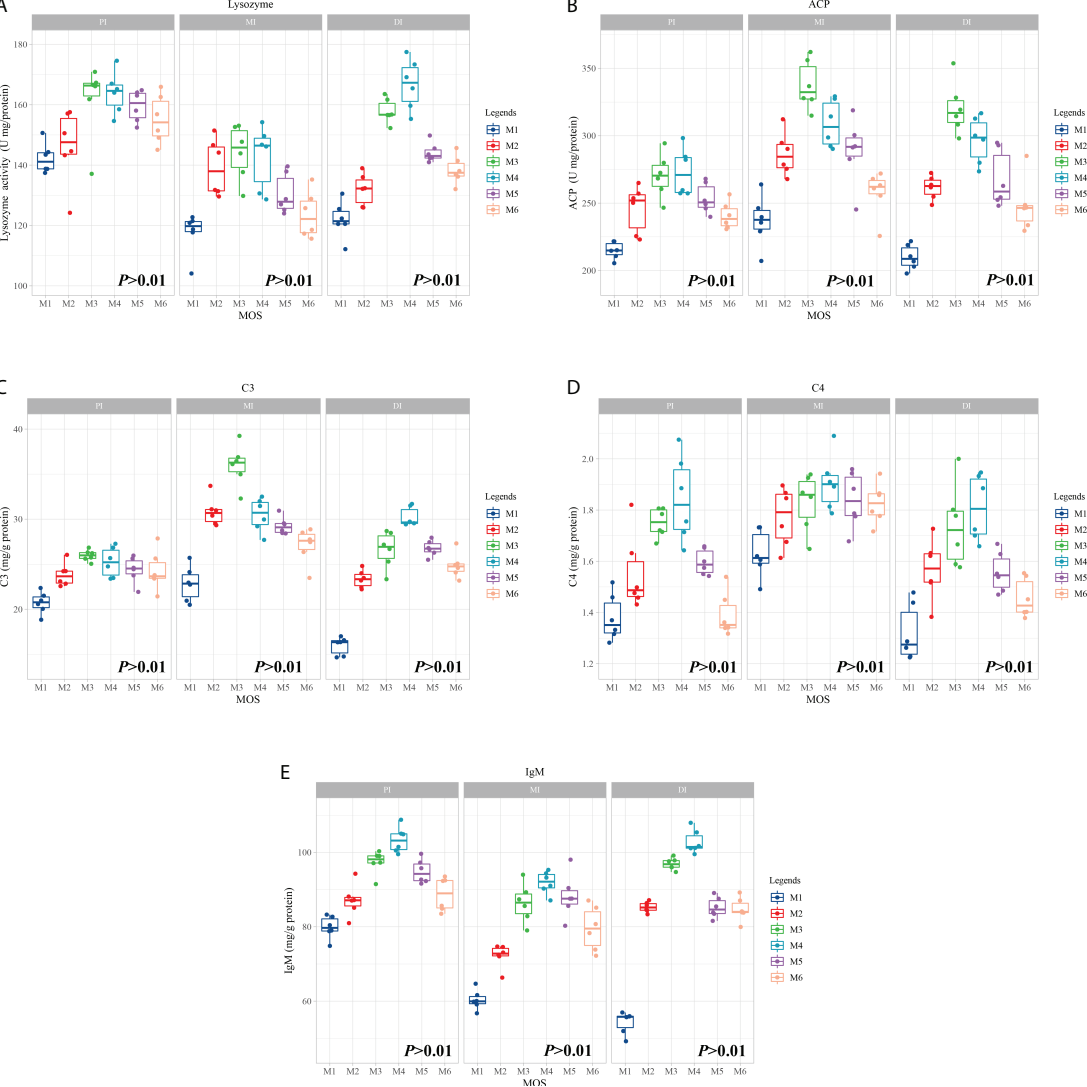
Effect of dietary MOS supplementation on immune barrier function in three intestinal segments of grass carp after infection of *Aeromonas hydrophila*. **(A–E)**, immune-related parameters, LZ, Lysozyme activity (U/mg protein); ACP, acid phosphatase (U/mg protein); C3, complement 3 (mg/g protein); C4, complement 4 (mg/g protein); IgM, immunoglobulin M (mg/g protein). N = 6 for each MOS level, different letters. The quadratic effects of MOS supplementation were assessed by using orthogonal polynomial contrasts.

To investigate the effects of MOS on fish intestines and phenotype after infection of *A. hydrophila*, we tested several major immunological parameters. **Figure 1** shows observations of immunological parameters. LZ, ACP activities, C3, C4 and IgM contents showed a quadratic effect in response to MOS supplementation (*P* < 0.01). In comparison with the control, while the MOS supplementation levels increased to 400, 600, 400, 400, and 400 mg/kg diet, in the PI, the LZ and ACP activities, C3, C4, and IgM contents reached their maximum levels, and with higher MOS addition, all of them showed a decreased trend; while the MOS supplementation levels increased to 400, 400, 400, 600, and 600 mg/kg diet, in the MI, then all of them (except C4 contents) showed a decreased trend; while the MOS supplementation levels increased to 600, 400, 600, 600, and 600 mg/kg diet, in the DI, the LZ and ACP activities, C3, C4, and IgM contents reached their maximum levels, and with higher MOS addition, all of them showed a decreased trend.

### Intestinal antimicrobial peptide-related gene expression

**Figure 2:**
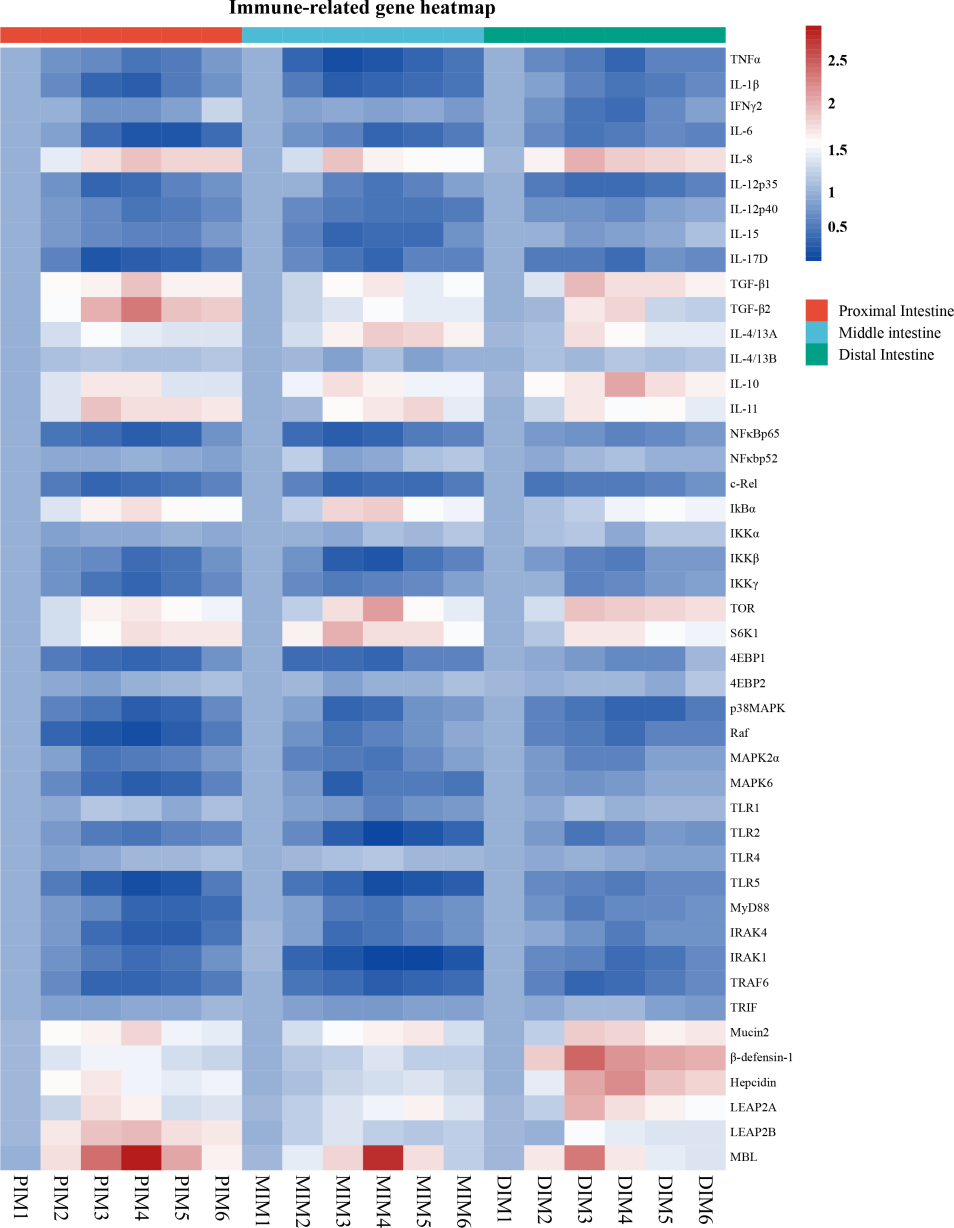
Heatmap of MOS supplementation changed expression of immune-related parameters in three intestinal segments of grass carp after infection of *Aeromonas hydrophila*. The signal values of up-regulation (red) and down-regulation (blue) were expressed and ranged from 0.5 to 2.5 folds.

To determine whether MOS affects the antimicrobial peptides in different segments, the expression of antimicrobial peptide-related genes was systematically measured, and the results are shown in **Figure 2**. The present study showed exhibited that antimicrobial peptide expression in all segments increased quadratically with MOS increasing levels (*P* < 0.05). Gene expression changes (fold-change) in the PI as compared to the control were as follows: MOS-400 mg/kg showed up-regulation of β-defensin-1 (0.46), Hepcidin (0.71) and LEAP2A (0.73) (*P* < 0.05), while MOS-600 mg/kg showed up-regulation of Mucin2 (0.8), LEAP2B (0.96) and MBL (1.95) (*P* < 0.05). In the MI, MOS-400 mg/kg showed up-regulation of LEAP2B (0.38) (*P* < 0.05) and MOS-600 mg/kg showed up-regulation of β-defensin-1 (0.39) (*P* < 0.05), MOS-600 mg/kg showed up-regulation of Mucin2 (0.68), Hepcidin (0.35), LEAP2A (0.64), and MBL (1.78) (*P* < 0.05). In the DI, MOS-400 mg/kg showed up-regulation of Mucin2 (1.88), β-defensin-1 (1.49), LEAP2A (1.00), LEAP2B (0.49) and MBL (1.34) (*P* < 0.05), while MOS-600 mg/kg showed up-regulation of Hepcidin (1.24) (*P* < 0.05).

### Intestinal inflammatory cytokines-related gene expression

To explore the influence of MOS on intestinal immune response, multiple intestinal inflammatory cytokines-related genes were investigated. As shown in **Figure 2**, inflammatory cytokines (including pro- and anti-inflammatory cytokines) expression in the PI (except IL-4/13B), MI (except IFNγ2, IL-4/13A and IL-4/13B) and DI (except IL-4/13B) increased quadratically with increasing MOS levels (*P* < 0.05). The maximum fold change in inflammatory cytokines in the PI as compared to control was as follows: MOS-400 mg/kg showed down-regulation of IFNγ2 (0.26), IL-12p35 (0.57) and IL-17D (0.70) and up-regulation of IL-11 (0.93) and IL-4/13A (0.55) (*P* < 0.05); MOS-600 mg/kg showed down-regulation of TNFα (0.47), IL-1β (0.61), IL-12p40 (0.46) and IL-15 (0.38) and up-regulation of IL-8 (0.95), IL-10 (0.73), TGF-β1(0.93) and TGF-β2 (1.37) (*P* < 0.05); MOS-800 mg/kg showed down-regulation of IL-6 (0.71) (*P* < 0.05). In the MI, MOS-400 mg/kg showed down-regulation of TNFα (0.74) and IL-1β (0.6) and up-regulation of IL-8 (0.92) and IL-10 (0.76) (*P* < 0.05); MOS-600 mg/kg, showed down-regulation of IL-6 (0.58), IL-12p35 (0.47) and IL-17D (0.56) (*P* < 0.05); MOS-800 mg/kg showed down-regulation of IL-12p40 (0.48) and IL-15 (0.58) and up-regulation of TGF-β1 (0.68), TGF-β2 (0.53), IL-4/13A (0.89) and IL-11 (0.81) (*P* < 0.05). In the DI, MOS-400 mg/kg showed down-regulation of IL-6 (0.46), IL-12p35 (0.55) and IL-15 (0.2) and up-regulation of IL-8 (1.05), TGF-β1 (0.97), IL-4/13A (0.76) and IL-11 (0.69) (*P* < 0.05); MOS-600 mg/kg showed down-regulation of TNFα (0.58), IL-1β (0.47), IFNγ2 (0.54), IL-12p40 (0.28) and IL-17D (0.53) and up-regulation of IL-10 (1.07) and TGF-β2 (0.83) (*P* < 0.05). Hance, the three intestinal segments were significantly influenced by dietary MOS supplementation in terms of decreased pro-inflammatory cytokine gene expression and increased anti-inflammatory cytokine expression. However, MOS supplementation did not affect IL-4/13B (in the PI, MI and DI) and IFNγ2 (only in the MI).

### Intestinal immunomodulatory key signaling molecules

To explore the influence of MOS on the regulative pathway of the intestinal inflammatory response, immunomodulatory key signaling molecule-related genes were investigated. As seen in **Figure 2**, the present study has found that intestinal immune response is closely related to NFκB and TOR signalling pathways after MOS supplementation. A quadratic relationship was found with increasing MOS levels (*P* < 0.05) in the NFκB and TOR signalling pathways-related molecules gene expression in the PI (except NFκBp52, IKKα, 4EBP2, TLR1, TLR4 and TRIF), MI (except NFκBp52, IKKα, 4EBP2, Raf, TLR4 and TRIF) and DI (except NFκBp52, IKKα, 4EBP2, TLR1, TLR4 and TRIF). The maximum fold change in signal molecular in the PI as compared to control was as follows: MOS-400 mg/kg showed down-regulation of c-Rel (0.58) and MAPK2α (0.47) (*P* < 0.05); MOS-600 mg/kg showed down-regulation of NFκBp65 (0.61), IKKβ (0.50), IKKγ (0.57), 4EBP1 (0.54), p38MAPK (0.62), Raf (0.73), MAPK6 (0.63), TLR2 (0.44), TLR5 (0.73), IRAK4 (0.63), IRAK1 (0.54) and TRAF6 (0.59) (*P* < 0.05); MOS-800 mg/kg showed down-regulation of MyD88 (0.58) (*P* < 0.05); MOS-600 mg/kg showed up-regulation of IκBα (0.74), TOR (0.73) and S6K1 (0.79) (*P* < 0.05). In the MI, MOS-400 mg/kg showed down-regulation of NFκBp65 (0.61), c-Rel (0.56), IKKγ (0.38), p38MAPK (0.58), Raf (0.43), MAPK6 (0.65) and IRAK4 (0.49) (*P* < 0.05); MOS-600 mg/kg showed down-regulation of IKKβ (0.57), 4EBP1 (0.56), MAPK2α (0.47), TLR1(0.32), TLR2 (0.81), TLR5 (0.72), MyD88 (0.45) and TRAF6 (0.66) (*P* < 0.05); MOS-800 mg/kg showed down-regulation of IRAK1 (0.82) (*P* < 0.05); MOS-400 mg/kg showed up-regulation of S6K1 (1.03) (*P* < 0.05); MOS-600 mg/kg showed up-regulation of IκBα (0.86) and TOR (1.14) (*P* < 0.05). In the DI, MOS-200 mg/kg showed down-regulation of c-Rel (0.49) (*P* < 0.05); MOS-400 mg/kg showed down-regulation of IKKγ (0.35), 4EBP1 (0.33), MAPK2α (0.35), MAPK6 (0.22), TLR2 (0.43), MyD88 (0.40) and TRAF6 (0.60) (*P* < 0.05); MOS-600 mg/kg showed down-regulation of NFκBp65 (0.37), IKKβ (0.43), p38MAPK (0.60), Raf (0.51), TLR5 (0.40), IRAK4 (0.39) and IRAK1 (0.53) (*P* < 0.05); MOS-400 mg/kg showed up-regulation of TOR (0.93) and S6K1 (0.71) (*P* < 0.05); MOS-800 mg/kg showed up-regulation of IκBα (0.53) (*P* < 0.05). However, MOS supplementation did not affect NFκBp52, IKKα, 4EBP2, TLR4 and TRIF in the PI, MI and DI, as well as TLR1 in the PI and DI.

### Correlation analysis

**Figure 3:**
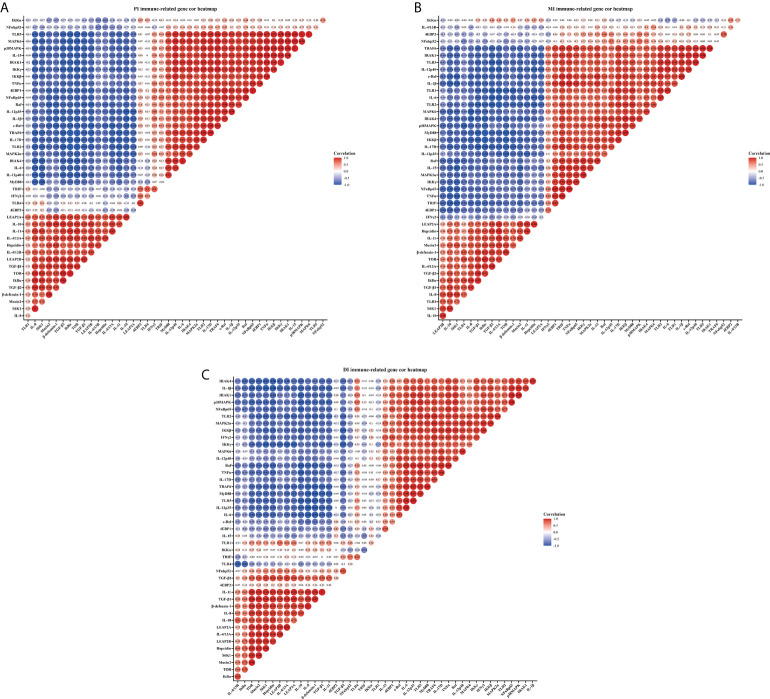
Correlation analysis of parameters in three intestinal segments of grass carp after infection of *Aeromonas hydrophila*. Proximal intestine **(A)**, Middle intestine **(B)**, and Distal intestine **(C)** of grass carp. R > 0.7, strong correlation; 0.5 < R < 0.7, moderate correlation; R < 0.5, weak correlation.

Correlation analysis was performed to examine the relationship between the expression of immune-related genes involved in intestinal immune response and the signal molecules involved in regulation (**Figure 3**). These data showed that the studied antimicrobial peptides have a strong and moderate negative correlation with studied anti-inflammatory cytokines mRNA levels, but a strong and moderate positive correlation with studied pro-inflammatory cytokines mRNA levels (*R* > 0.7, strong correlation; 0.7 > *R* > 0.5, moderate correlation; *R* < 0.5, weak correlation) in the three intestinal segments. Gene expression of the majority of studied pro-inflammatory cytokines revealed a strong and moderate positive correlation with NFκBp65 mRNA levels, whereas the majority of studied anti-inflammatory cytokines had a strong and moderate positive correlation with TOR gene expression in the three intestinal segments (except IL-4/13B in the MI and IL-15 in the DI). In addition, the mRNA levels of TLR2 and TLR5 showed a strong and moderate positive correlation with a majority of pro-inflammatory cytokines in the three intestinal segments.

### Immunohistochemical analysis of key role protein

**Figure 4:**
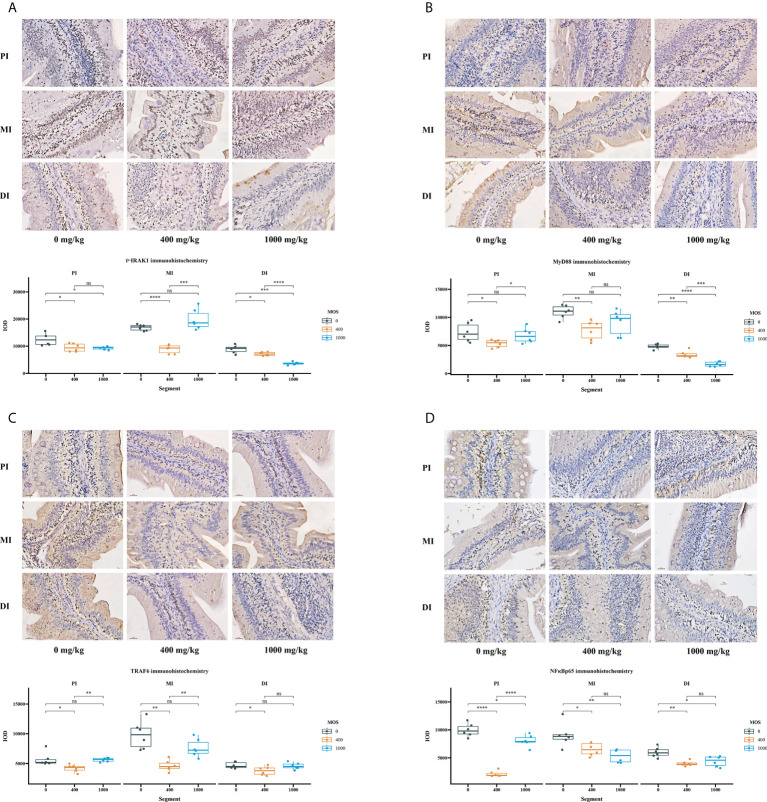
The effect of MOS on p-IRAK1, MyD88, TRAF6, and NFκBp65 expression by immunohistochemistry method in three intestinal segments after infection of *Aeromonas hydrophila*. **(A)** p-IRAK1 protein expression, **(B)** MyD88 protein expression, **(C)** TRAF6 protein expression, **(D)** NFκBp65 protein expression; Quantification of the positive area as revealed by Image Pro Plus 6.0. N = 6 for each MOS level. Differences among the variables were assessed using Student’s t-tests. Statistical significance: *p < 0.05; **p < 0.01, ***p < 0.001, ****p < 0.0001; ^ns^ *p* > 0.05.

Immunohistochemical analysis was performed after the bacterial challenge to observe how MOS supplementation affected intestinal epithelium (IRAK1, MyD88, TRAF6 and NFκBp65) in the three intestinal segments (**Figure 4**). The IOD of p-IRAK1, MyD88, TRAF6 and NFκBp65 were quantified using Image Pro Plus 6.0 software. In comparison with the control, in **Figure 4A**, the p-IRAK1 IOD displayed a declining trend among all intestinal tracts (*P* < 0.05) for the MOS-400 mg/kg group, but only in the PI and MI (*P* < 0.05) for the MOS-1000 mg/kg group, with the p-IRAK1 IOD in the MI not having obvious changes (*P* > 0.05); in **Figure 4B**, while the MyD88 IOD of MOS-400 mg/kg group displayed a declining trend among all intestinal tracts (*P* < 0.05), the MyD88 IOD of 1000 mg/kg group was only significantly decreased in the DI (*P* < 0.05), with no obvious changes in the PI and MI (*P* > 0.05); in **Figure 4C**, the TRAF6 IOD of MOS-400 mg/kg group decreased among all intestinal tracts (*P* < 0.05) but there was no significant difference for the 1000 mg/kg group (*P* > 0.05); in **Figure 4D**, the NFκBp65 IOD of the 400 mg/kg group and 1000 mg/kg group showed a declining trend among all intestinal tracts (*P* < 0.05).

### Key role protein levels of intestinal immune response

**Figure 5:**
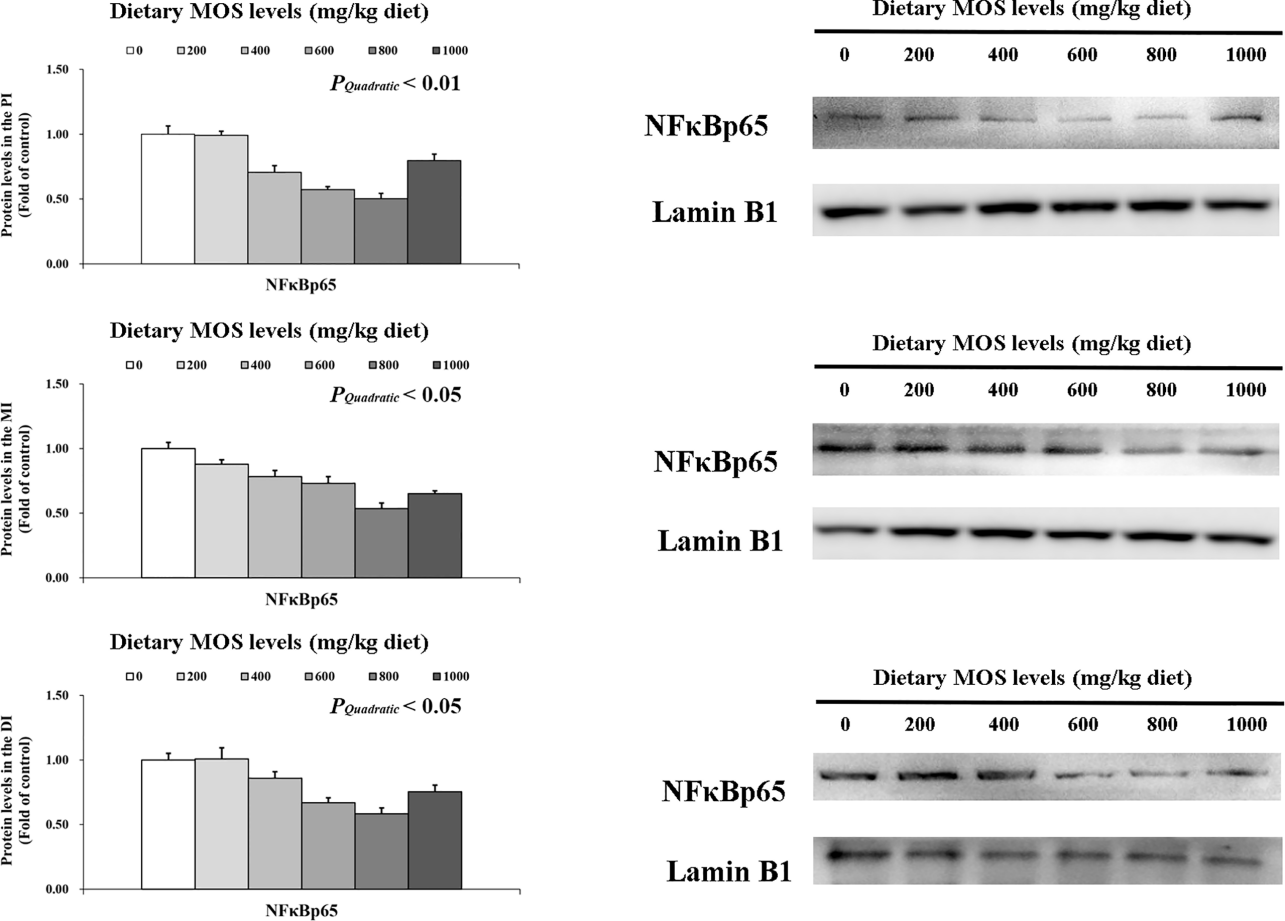
Western blot analysis of NFκBp65 protein levels in three intestinal segments of grass carp after infection of *Aeromonas hydrophila*. Data represent means of three fish in each group, error bars indicate S.D. The quadratic effects of MOS supplementation were assessed by using orthogonal polynomial contrasts.

**Figure 6:**
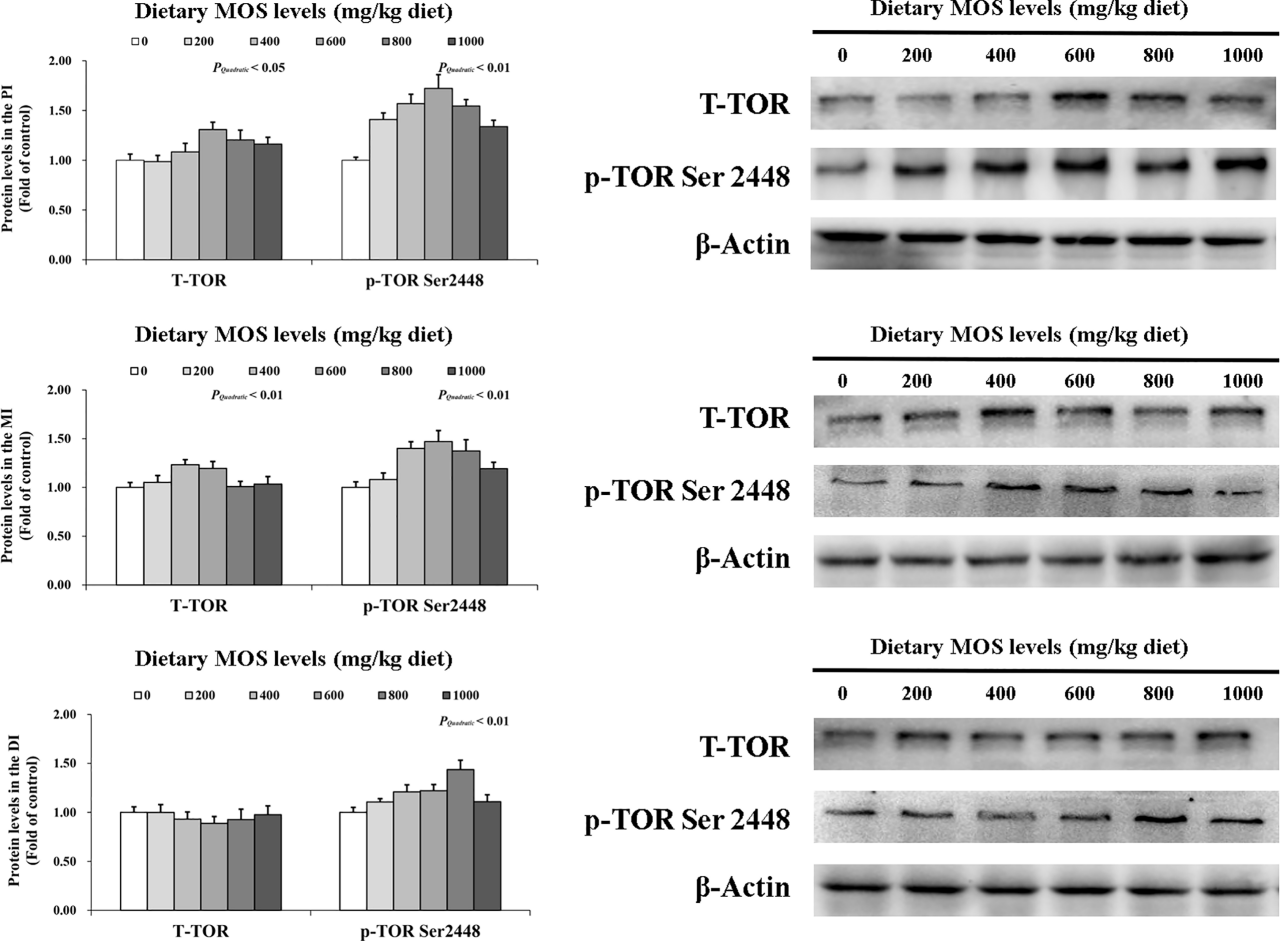
Western blot analysis of T-TOR and p-TOR Ser2448 protein levels in three intestinal segments of grass carp after infection of *Aeromonas hydrophila*. Data represent means of three fish in each group, error bars indicate S.D. The quadratic effects of MOS supplementation were assessed by using orthogonal polynomial contrasts.

**Figure 7:**
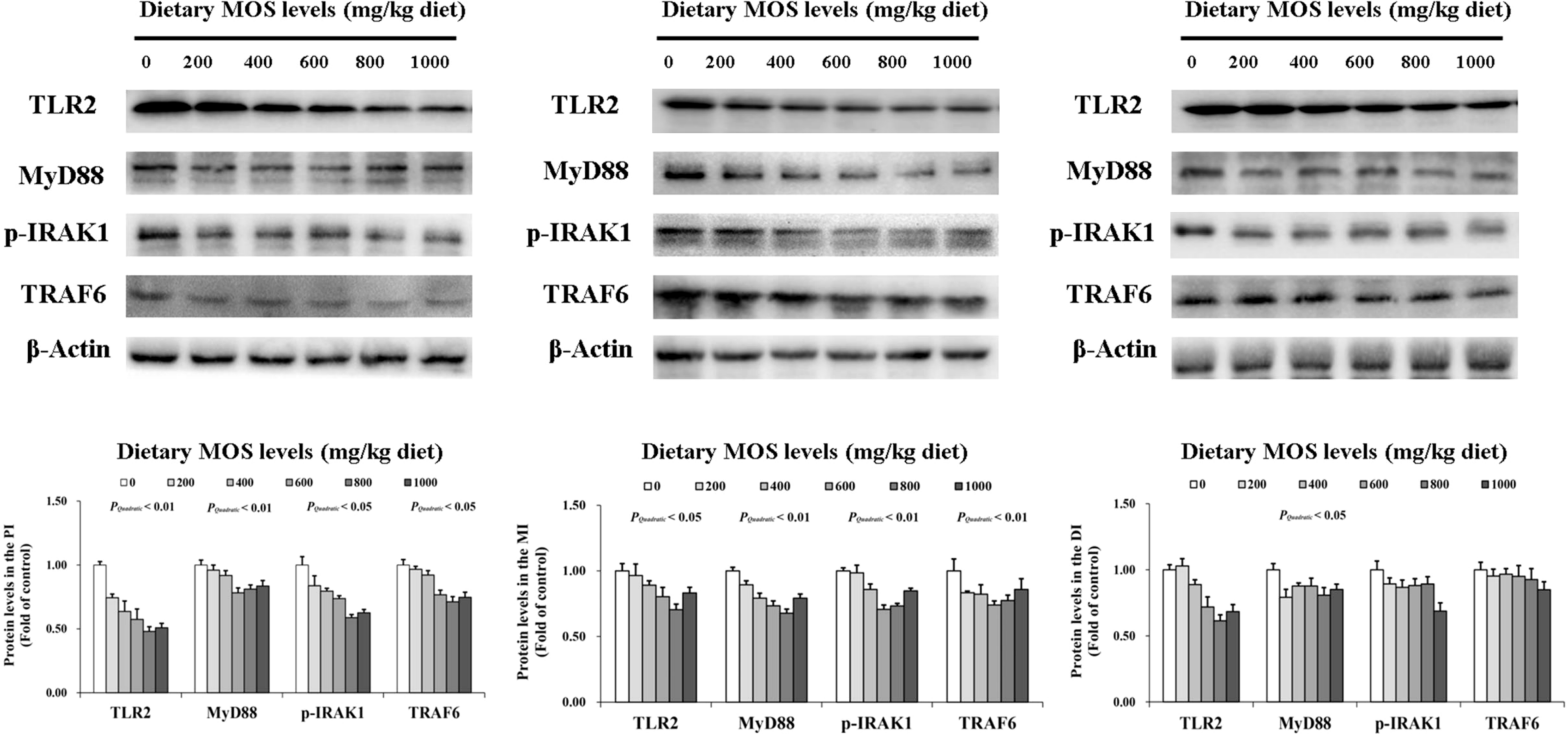
Western blot analysis of TLR2, MyD88, p-IRAK1, and TRAF6 protein levels in three intestinal segments of grass carp after infection of *Aeromonas hydrophila*. Data represent means of three fish in each group, error bars indicate S.D. The quadratic effects of MOS supplementation were assessed by using orthogonal polynomial contrasts.

To determine how MOS affects the immune response in the intestine, several crucial signalling molecules (TOR, NFκBp65, TLR2, MyD88, IRAK1, and TRAF6) were investigated, respectively (**Figures 5**–**7**). In **Figure 5**, in comparison with the control, the NFκBp65 protein levels declined with MOS addition 800 mg/kg among all intestinal segments, and then gradually increased. In **Figure 6**, the p-TOR Ser 2448 protein levels in the PI, MI, and DI increased quadratically with increasing MOS levels (*P* < 0.05), while the T-TOR protein levels only increased quadratically in the PI and MI. In compare to the control, the p-TOR Ser 2448 protein levels were elevated with MOS addition (600(PI), 600(MI), and 800(DI) mg/kg) for all intestinal segments, before gradually decreased. As shown in **Figure 7**, with increasing levels of MOS, the MyD88 protein levels in the PI, MI and DI decreased quadratically (*P* < 0.05), and so did protein levels of TLR2, p-IRAK1 and TRAF6 in the PI and MI. Compared with the control group, the TLR2 protein levels declined with MOS supplementation up to 800 mg/kg in the PI, MI and DI, and then plateaued; the MyD88 protein levels declined with MOS supplementation up 600, 800, and 200 mg/kg in the PI, MI and DI, and then plateaued; the p-IRAK1 protein levels were declined with MOS supplementation up to 800, 600, and 1000 mg/kg in the PI, MI and DI respectively, and then plateaued (except in the DI); the TRAF6 protein levels declined with MOS supplementation up to 800, 600 and 1000 mg/kg in the PI, MI, and DI respectively, and then plateaued (except in the DI).

## Discussion

The current study was based on the same growth trial used in our previous work in grass carp, which was part of a larger research to investigate the protective effect of MOS supplementation on fish intestinal barrier function (28). As prebiotic zootechnical feed ingredients, MOS derived from *Saccharomyces cerevisiae*’s outer cell wall has been extensively studied to replace antibiotics as a growth promoter and immunopotentiator (2). Among aquatic animals, *A. hydrophila* is known for causing severe enteritis and damage to intestinal structures, which could be hazardous to intestinal health (31). According to our previous research, fish growth, intestinal antioxidant capacity, disease resistance, and structural integrity can be enhanced by optimal MOS addition after *A. hydrophila* infection (6, 28). Many researchers consider fish growth and development to be strongly correlated with intestinal barrier function at the moment (32, 33). Nevertheless, the intestinal immune barrier functions also a crucial role in maintaining normal intestinal barrier function (34). As we previously demonstrated, MOS can enhance intestinal antioxidant capacity and structural integrity when confronted with *A. hydrophila* (6, 28). However, these evidences do not provide a sufficient understanding of MOS’s protection of intestinal barriers. Consequently, based on the previous works on physical barriers (antioxidant ability and structural integrity), we carried out further exploration of immune barriers and revealed potential molecular mechanisms. Firstly, we investigated the effects of MOS supplementation on antibacterial substances in the intestine of grass carp.

### MOS promotes the production of antibacterial substances

As important physical and immune barriers, intestinal epithelial cells (IECs) prevent microbial communities from spreading from the lumen into other parts of the body (35). There is a close relationship between the fish immune barrier and antimicrobial substances, such as antimicrobial compounds and antimicrobial peptides. To our knowledge, similar to mammals, the IECs of bony fish are composed of multiple types of cells with different functions such as goblet cells, Paneth cells, absorptive enterocytes, enteroendocrine cells and so on (35, 36). The first line of defense against microbial invasion is the secretion of glycosylated mucins (Mucin2) from goblet cells to the intestinal tract (37). Other antibacterial substances such as lysozyme and antimicrobial peptides are secreted by Paneth cells to remove and kill pathogenic microorganisms exogenously (38), whereas ACP involves localization in the enterocytes to protect intestinal epithelial cells and releases superficial cells for sloughing (39). Additionally, the complement system recognizes pathogenic microorganisms and rapidly responds with a tailored defense response (40). IECs have been identified as primary producers of complement components across the gastrointestinal tract (41), whereas IgM is the only antibody that responds to pathogens in both the systemic and mucosal compartments, produced by lymphocytes in fish intestines (42).

The current study showed that the MOS-400 mg/kg group elevated LZ and ACP activities, and the C3, C4, and IgM contents, as well as up-regulated hepcidin, LEAP2A, LEAP2B, Mucin2 and β-defensin-1 mRNA levels in all three intestinal segments of grass carp. These data suggested that dietary MOS supplementation promotes the production of antibacterial substances in the intestine of fish. Our previous work has confirmed that different degrees of pathological changes (such as hyperaemia, edema, inflammatory cell infiltration, cell necrosis, etc.) occurred in the intestine of grass carp after being challenged by *A. hydrophila* (28). This indicated that following pathogen infection, the intestinal structural integrity was compromised, and excessive inflammation was triggered, which eventually resulted in IECs necrosis. As a result of the necrosis of IECs, many cells (such as enterocytes, goblet cells, Paneth cells, etc.) will lose their immune function (35, 43). We speculated that MOS could increase the content of antibacterial substances in the intestinal tract through multiple pathways. One such way was that, MOS would bind to pathogenic bacteria and prevent them from colonizing intestinal epithelium, thus protecting IECs. This was consistent with our previous results, in which the *A. hydrophila* population was significantly reduced and intestinal ultrastructure and apical junction complex were significantly improved (6, 28). Another possibility could be that MOS may activate the complement lectin pathway to promote a series of cascade reactions and eventually form a membrane-attacking complex to effectively kill pathogenic microorganisms (40). As we know, molecular patterns (MAMPs) that are associated with microbial growth include mannose-binding lectins (MBLs) that recognize mannose residues on cell surfaces (40). During pathogen invasion, MBL binds to mannose residues on pathogen surfaces and further activates the complement system (41). Our hypothesis was supported by the present study that showed that the expression levels of MBL were significantly up-regulated.

What is noticeable is that the optimum level for most antimicrobial substances is between 400 and 600 mg/kg MOS, before a plateau or gradual declines was observed with greater MOS supplementation. There may be an excessive effect caused by the excessive addition of MOS. It has been reported that MOS supplementation could increase the number of goblet cells in European sea bass (24). Another study has confirmed that abnormal goblet cell proliferation can produce excess mucus, leading to the occurrence of inflammation in LS174T cells (44). Our results also showed that the expression of the inflammatory cytokines in the high dose MOS group (1000 mg/kg) was significantly higher than that of the more optimal MOS groups (400-600 mg/kg), indicating that excessive MOS is not conducive to the recovery of intestinal homeostasis after the challenge. Although we have studied the effect of MOS on multiple antibacterial substances after the *A. hydrophila* challenge, more representative evidence of the immune barrier is needed to support it. As described above, the intestinal immune barrier is also closely associated with the inflammation response, which is primarily mediated by inflammatory cytokines (7, 15). Consequently, we next investigated the relationship between dietary MOS supplementation and the inflammatory cytokines in the intestine of grass carp.

### MOS attenuates the inflammatory response

The cellular components of the intestinal immune barrier include not only the largest proportion of IECs in the superficial layer (mucosal layer), but also the immune cells (such as macrophages, dendritic cells, adaptive, lymphocyte cells, etc.) with special functions in the inner layer (lamina propria) (35). When pathogens penetrate the mechanical barrier of the intestinal wall (including mucus layer, epithelium, and Apical junction complex), a secondary line of defense is initiated (immune cell dominance), triggering an immune response (45). Although almost all intestinal cells can secrete various cytokines, in bony fish, immune cells play a major role (7, 36). After receiving external and internal signals, immune cells contribute to the immune response by secreting pro-inflammatory cytokines (such as TNFα, IL-β, IL-6, and IL-8) and anti-inflammatory cytokines (such as TGF-β and IL-10) (15, 46). It was confirmed that down-regulating pro-inflammatory cytokines and up-regulating anti-inflammatory cytokines mRNA levels in fish could reduce excessive inflammatory response (46). In the current study, by examining the mRNA levels of multiple studied inflammatory cytokines, the data from all three intestinal segments showed that almost all of the studied pro-inflammatory cytokines were down-regulated (except INFγ2 in the MI) with optimal MOS supplementation (400-800 mg/kg), whereas studied pro-inflammatory cytokines anti-inflammatory cytokines were up-regulated (except IL-4/13B in all three segments) with optimal MOS supplementation (400-600 mg/kg). Similarly, MOS reduced TNFα and IL-6 expression in the intestine after *A. hydrophila* challenged in Blunt Snout Bream (*Megalobrama amblycephala*) (47). Thus, we believe that MOS has a positive prebiotic effect on the fish intestinal tract.

Interestingly, MOS supplementation could up-regulate IL-8 expression in all three intestinal segments. To our knowledge, IL-8 is a typical proinflammatory cytokine that is involved in the inflammatory response (48). This result, contrary to our expectations, seems to suggest an increase in local inflammation caused by MOS supplementation. Coincidentally, the results are not unique. It has been reported that MOS up-regulated IL-8 expression in the intestine after *A. hydrophila* challenged in Nile Tilapia (*Oreochromis niloticus*) (49). Another study also showed that the mRNA levels of IL-8 was up-regulated with MOS supplementation after infection (3). Although our results are consistent with these studies, there is no reasonable explanation for this abnormal phenomenon yet. Thus, we make the following assumptions based on some reliable literature. The up-regulation of IL-8 from MOS supplementation in the three intestinal segments of grass carp might be partially related to prostaglandins (PGs). A study on the European sea bass indicated that MOS could increase PGs content in the intestine (24). It was reported that PGs could up-regulate IL-8 mRNA levels in the human colonic epithelial cell (50), which may partly support our hypothesis. It is worth noting that MOS supplementation had no effect on the mRNA levels of IL-4/13B in the three intestinal segments of grass carp. A possible reason for this difference could be partially related to phosphorus digestibility. A study showed that MOS supplementation could increase phosphorus digestibility in the ileum of piglets (18). A previous study from our laboratory also confirmed that phosphorus does not affect IL-4/13B mRNA levels in the head kidney and spleen of grass carp (19), which may partly support our hypothesis. In addition, our data also showed that MOS had no significant effect on INFγ2 in the MI segments. However, existing literature is unable to provide any reasonable explanation, thus subsequent studies are needed to further explore and verify this. It needs to be emphasized that most of the inflammatory cytokines involved in the inflammatory response are typically modulated by classical immune-related signalling pathways (20, 21, 26). Thus, we next investigated the relationships between the dietary MOS supplementation and the immuno-related signalling pathway in the intestine of grass carp.

### MOS regulates signalling pathways in inflammatory responses

In mammals, the mRNA levels of pro-inflammatory cytokines are regulated by IKK (including IKKα, IKKβ and IKKγ), which could suppress IκBα degradation, and then inhibit activation of NFκB (including NFκBp52, NFκBp65 and c-Rel) (51). In addition, in human PBMCs, the study showed that mTOR enhances the anti-inflammatory cytokine IL-10 activity (52). It was reported that mTOR could phosphorylate S6K1 and inhibits the initiation factor 4EBP to initiate the translation of distinct mRNAs in mammals (53). Based on the current research progress and the results of previous studies from our laboratory, it is certain that the function of these key inflammatory signalling molecules between fish and mammals is conserved (54). However, there are no reports on prebiotics involving signal molecules related to intestinal immune regulation of fish. To fill this gap in knowledge, we investigated gene expression and protein levels of key signalling molecules in the TOR and NFκB signalling pathways.

Our results found that optimal MOS supplementation down-regulated gene expression of NFκBp65, c-Rel, IKKβ and IKKγ, and it up-regulated IκBα gene expression in all three intestinal segments. Further correlation analysis showed that these pro-inflammatory cytokines (except IFN-γ2 in the MI) gene expression were positively correlated with NFκBp65 and c-Rel mRNA levels, but had a negative correlation with IL-8 mRNA levels in the three intestinal segments. Furthermore, the mRNA levels of signal molecules NFκBp65, c-Rel, IKKα, IKKβ and IKKγ were negatively correlated with IκBα mRNA levels. In addition, protein levels of NFκBp65 in the three intestinal segments were significantly reduced with MOS supplementation, which further indicated that the NFκB pathway was effectively inhibited. These results indicate that MOS supplementation down-regulated the mRNA levels of the studied pro-inflammatory cytokines (except *IL-8* and IFN-γ2 (in the MI)), which may be partly related to (IKKβ and IKKγ)/IkBα/(NFκBp65 and c-Rel) signalling pathway in the three intestinal segments of fish. Interestingly, we found that dietary MOS supplementation did not affect NFκBp52 and IKKα mRNA levels in the three intestinal segments of grass carp. There could be several possible reasons for these differences. First, MOS supplementation did not affect NFκBp52 mRNA levels and this might be partially associated with IKKα levels also not being affected. A study on human lung carcinoma cells indicated that IKKα could activate NFκBp52 mRNA levels (55). In the present study, since MOS supplementation did not affect IKKα in the intestine, this may partially support our speculation. Second, MOS supplementation did not affect IKKα mRNA levels possibility due to its association with *E. coli* and PKC-ζ. Our previous work showed that MOS could decrease the number of *E.coli*, which are mainly located in the intestinal tract (28). In human HT-29/B6 cells, it has been reported that *E.coli* could increase the protein level of PKC-ζ (56). Another study on rat Kupffer cells showed that PKC-ζ could activate IKKβ and IKKγ but had no effect on IKKα expression (57), thus possibly explaining our observations. In addition, our results showed that studied anti-inflammatory cytokines were up-regulated and positively correlated with TOR mRNA levels with MOS supplementation. Furthermore, the protein levels of p-TOR Ser 2448 in the three intestinal segments were significantly increased with MOS supplementation, which further indicates that the TOR pathway was effectively activated. This result is consistent with the other study in juvenile hybrid grouper intestine that showed up-regulation of TLR3 with MOS supplementation (27). One interesting aspect is that dietary MOS supplementation could down-regulate mRNA levels of 4EBP1 but not 4EBP2 in three intestinal segments, which might be partly due to butyrate and p38MAPK. A study on weaned rabbits indicated that MOS could increase butyrate content in the caecal (58). A previous study from our lab confirmed that butyrate could activate p38MAPK (23). It was reported in humans that activation of p38 MAPK could down-regulate the expression of 4EBP1 but have no effect on the expression of 4EBP2 (59), thus partially supportting our hypothesis. These results indicate that MOS supplementation up-regulated anti-inflammatory cytokines (except 4EBP2) mRNA levels, which may be partly related to TOR/S6K1/4EBP (not 4EBP2) in the three intestinal segments.

In light of the above evidence, it has been established that MOS regulates inflammation primarily by inhibiting the NFκB and TOR pathways. Nevertheless, the mechanism by which mannan oligosaccharides activate these classical pathways is unknown. As far as we know, molecular patterns associated with pathogens (PAMPs) could be recognized by PRRs and consequently stimulate immunity and defense (60). As one of the classic PRRs of immune activation, TLRs have become a focal point of immune response research, which could activate NFκB by a cascade of reactions (26). Therefore, we focused on pattern recognition receptors and selected several TLRs (TLR1, TLR2, TLR4 and TLR5) that are closely related to pathogen interaction for preliminary exploration. The present data displayed that MOS supplementation down-regulated TLR1 (in the MI), TLR2, TLR5 and the downstream related signal molecules (MyD88, IRAK1, IRAK4 and TRAF6) in the three intestinal segments. The results of correlation analysis also show that the TLRs signalling pathway was positively correlated to most of the studied pro-inflammatory cytokines. Our results are similar to the previous study in Ussuri catfish (*Pseudobagrus ussuriensis*), which showed that in the intestine, the gene expression of TLR2 and MyD88 were down-regulated with yeast culture (the main component is MOS) supplementation after being *A. hydrophila* challenged (61). Interestingly, some previous studies involving growth trials of other animals yielded different findings, for instance, in juvenile hybrid grouper, TLR3 was up-regulated (27), and in chickens, both TLR2 and TLR4 gene expressions were up-regulated following MOS supplementation (62). We speculate that the main possible reason for this interesting phenomenon is related to challenge stress. Both studies on hybrid grouper and chickens did not involve a bacterial challenge, whereas the current experiment involved on. Based on previous studies, we found that MOS is an excellent immunostimulant, and the optimal level of addition can maintain a sensitive immune status under normal circumstances, which were validated in multiple bony fish studies (2, 63). However, *A. hydrophila* usually causes excessive intestinal inflammation in fish (1), and our data suggest that MOS supplementation can reduce such a symptom in the intestine of fish. In addition, our study also found that MOS had no significant effect on TLR4 in the three intestinal segments. These results were beyond our expectations. Generally, TLR4 plays a key role in intestinal pattern recognition (recognizing bacteria lipopolysaccharides) during bacterial infection in mammals (26). However, unlike mammals, TLR4 does not recognize LPS in fish (64). This may partly explain why TLR4 expression was not significant in the intestine. However, the specific mechanism involved is not clear, which is worthy of further study in future work. To our knowledge, as a downstream signal molecular, TRAF6 could activate the NFκB signalling pathway by activating IKKβ (26, 65). Our result also showed that TRAF6 has a strong correlation with IKKβ, IKKγ, and NFκB, which partly explains how MOS inhibits TLRs-mediated excessive inflammatory signalling. In conclusion, we believe that the inhibiting effect of TLRs pathway by MOS may be related to intestinal immune responses under *A. hydrophila* infected condition, but there are still some limitations to the current study, which requires further verification by more experiments. However, it is notable that the existing studies do not investigate the mechanism by which MOS affects TOR signals. A surprising finding has been that PI3K and Akt regulate TOR, and these molecules are also regulated by TLR signals (66). It is contradictory for MOS to inhibit TLRs signal and activate TOR signal. We hypothesized that since intestinal immune barrier function is the result of simultaneous interaction of multiple intracellular communication, there must be crosstalk between other signalling pathways of different signalling molecules leading to different signalling mechanisms. A study in weaned pigs showed that MOS could up-regulate GLUT-2 mRNA levels in the duodenum (67). Another study on mice showed that GLUT-2 has a cooperative mechanism with Akt/mTOR/S6K1 signalling pathway in pancreatic beta cells (68). Our results also showed MOS activated TOR signal, which partially supports our hypothesis. Nevertheless, we still lack an understanding regarding the mechanism. Therefore, we expect to use next-generation sequencing and cell culture-related technologies in the future to reveal how MOS regulates intestinal immune responses through specific subtypes and related signalling pathways in the TLRs family and TOR signal.

### Difference in immunological effect of MOS on different organs

We found several two interesting findings when comparing the results of intestinal immunological effect in this study to those of previous studies from our lab on the multiple functional organs (head kidney, spleen, and skin) (4, 5). The content of antimicrobial substances and the expression of inflammatory cytokines in the intestine showed a similar overall trend compared with the head kidney, spleen, and skin, but it was obvious that the content of LZ and ACP in the intestine was significantly lower than that in the head kidney and spleen. The reason for this phenomenon may be related to organ structure. To our knowledge, the head kidney, and spleen of fish contain a large number of immune cells (monocytes), which can produce a large number of antimicrobial substances (LZ and ACP) and inflammatory cytokines when immunologically stimulated (69, 70), which were supporting our hypothesis. Secondly, we also found that MOS had different effects on the expression level of IL-8 in different organs of grass carp after challenged, which was up-regulated in the intestine, insignificant in the head kidney, and spleen, and significantly down-regulated in the skin (4, 5). We speculate that the possible reason for this phenomenon is related to the specific expression of PGs in the intestine. As mentioned above, MOS can increase the intestinal PGs content of European sea bass (24), which can increase the expression of IL-8 in human colonic epithelial cells (50), thus supporting our hypothesis. In addition, studies on greater amberjack also found that different types of MOS could enhance disease resistance by regulating inflammatory cytokines in multiple organs under parasite infection conditions (3). However, this was not completely consistent with our results. In conclusion, we believe that the immunological response of MOS to different organs is related to many factors in fish, including fish species, feeding time, dosage, pathogen, and different structure of functional organs.

## Conclusions

Overall, this study presents clear evidence that dietary MOS enhanced grass carp immune barrier function after infection with *A. hydrophila*. Based on the results of our study, dietary MOS supplementation improves the immune barrier function of the intestine in several ways as follows: (1) dietary MOS supplementation could increase intestinal disease resistance, producing more antibacterial compounds and immunoglobulins. (2) dietary MOS supplementation could regulate the dynamic balance of inflammatory cytokines, inhibiting the excessive inflammatory response. (3) MOS supplementation improved fish immune barrier function may partly be related to modulating TLRs/MyD88/NFκB and TOR/S6K1/4EBP signalling pathways.

## Data availability statement

The datasets presented in this study can be found in online repositories. The names of the repository/repositories and accession number(s) can be found in the article/**Supplementary Material**.

## Ethics statement

The animal study was reviewed and approved by Animal Care Advisory Committee of Sichuan Agricultural University.

## Author contributions

Z-YL performed formal analysis, investigation and writing original draft. LF performed conceptualization, funding acquisition and supervision. W-DJ performed data curation, validation, project administration and writing review & editing. PW performed conceptualization, methodology, validation, data curation and project administration. YL and JJ performed project administration. S-YK, LT, S-WL, C-BZ performed resources. X-QZ performed conceptualization, designed experiment, supervision and funding acquisition. All authors contributed to the article and approved the submitted version.

## Funding

This research was financially supported by National Key R&D Program of China (2019YFD0900200 and 2018YFD0900400), National Natural Science Foundation of China for Outstanding Youth Science Foundation (31922086), the Young Top-Notch Talent Support Program, Supported by China Agriculture Research System of MOF and MARA (CARS-45), and supported by Sichuan Science and Technology Program (2019YFN0036). The authors would like to thank the personnel of these teams for their kind assistance.

## Conflict of interest

Authors S-YK, LT, S-WL, C-BZ were employed by Sichuan Animtech Feed Co. Ltd.

The remaining authors declare that the research was conducted in the absence of any commercial or financial relationships that could be construed as a potential conflict of interest.

## Publisher’s note

All claims expressed in this article are solely those of the authors and do not necessarily represent those of their affiliated organizations, or those of the publisher, the editors and the reviewers. Any product that may be evaluated in this article, or claim that may be made by its manufacturer, is not guaranteed or endorsed by the publisher.
