# Supplementary material for: Dietary mannan oligosaccharides strengthens intestinal immune barrier function via multipath cooperation during Aeromonas Hydrophila infection in grass carp (Ctenopharyngodon Idella)
Source: Front Immunol. 2022 Sep 13;13:1010221. doi: 10.3389/fimmu.2022.1010221 (PMC9513311; doi:10.3389/fimmu.2022.1010221)
Supplement: Supplementary file 2 [file DataSheet_2.docx]

Supplementary Material

**Supplementary Table 1. Composition and nutrient content of the diet.**

| Ingredients | % | Nutrient content | % |
| --- | --- | --- | --- |
| Fish meal | 7.80 | Crude protein ^4^ | 28.69 |
| Gelatin | 6.00 | Crude lipid ^4^ | 5.36 |
| Soybean protein concentrated | 26.00 | n-3 ^4^ | 1.04 |
| Corn starch | 19.90 | n-6 ^4^ | 0.96 |
| α-starch | 24.00 | Available phosphorus ^4^ | 0.40 |
| Fish oil | 2.34 |  |  |
| Soybean oil | 1.81 |  |  |
| Cellulose | 5.00 |  |  |
| Ca (H_2_PO4)_2_ | 1.30 |  |  |
| Vitamin premix ^1^ | 1.00 |  |  |
| Mineral premix ^2^ | 2.00 |  |  |
| MOS premix ^3^ | 1.00 |  |  |
| Choline chloride (50%) | 1.00 |  |  |
| Ethoxyquin (30%) | 0.05 |  |  |
| DL-Met (99%) | 0.61 |  |  |
| L-Trp (99%) | 0.08 |  |  |
| Thr (98.5%) | 0.11 |  |  |

^1^ Per kilogram of vitamin premix (g/kg): retinyl acetate (500,000 IU/g), 0.39; cholecalciferol (500,000 IU/g), 0.40; D, L-α-tocopherol acetate (50%), 23.23; menadione (22.9%), 0.83; cyanocobalamin (1%), 0.94; D-biotin (2%), 0.75; folic acid (95%), 0.42; thiamine nitrate (98%), 0.09; ascorhyl acetate (95%), 9.77; niacin (99%), 4.04; meso-inositol (98%), 19.39; Calcium-D-pantothenate (98%), 3.85; riboflavin (80%), 0.73; pyridoxine hydrochloride (98%), 0.62. All ingredients were diluted with corn starch to 1 kg. ^2^ Per kilogram of mineral premix (g kg^-1^): MnSO_4_⋅H_2_O (31.8% Mn), 2.6590; MgSO_4_⋅H_2_O (15.0% Mg), 200.0000; FeSO_4_⋅H_2_O (30.0% Fe), 12.2500; ZnSO_4_.H_2_O (34.5% Zn), 8.2460; CuSO_4_⋅5H_2_O (25.0% Cu), 0.9560; KI (76.9% I), 0.0650; Na_2_SeO_3_ (44.7% Se), 0.0168. All ingredients were diluted with corn starch to 1 kg. ^3^ MOS premix (mg kg^-1^): premix was added to obtain graded levels of MOS. ^4^ Crude protein and crude lipid content were measured value. Available phosphorus, n-3 and n-6 contents calculated according to NRC (2011).

**Supplementary Table 2. Biochemical index determination method and commercial kit.**

| Indices | Method | Source | Catalog No. |
| --- | --- | --- | --- |
| Acid phosphatase kit | Spectrophotometric method | Nanjing Jiancheng Bio-engineering Institute | A060-2-2 |
| Lysozyme kit | Spectrophotometric method | Nanjing Jiancheng Bio-engineering Institute | A050-1-1 |
| Complement 3 kit | Immunoturbidimetry | Yilikang Biotechnology Co (Zhejiang, China) | Y10025 |
| Complement 4 kit | Immunoturbidimetry | Yilikang Biotechnology Co (Zhejiang, China) | Y10026 |
| Immunoglobulin M kit | Immunoturbidimetry | Yilikang Biotechnology Co (Zhejiang, China) | Y10024 |

**Supplementary Table 3. Real-time PCR primer sequences ^1^**

| **Target gene** | **Primer sequence (5’→3’)** | **Temperature(°C)** | **Accession number** |
| --- | --- | --- | --- |
| **hepcidin** | **Forward:** AGCAGGAGCAGGATGAGC | 59.3 | JQ246442.1 |
|  | **Reverse:** GCCAGGGGATTTGTTTGT |  |  |
| **LEAP2A** | **Forward:** TGCCTACTGCCAGAACCA | 59.3 | FJ390414 |
|  | **Reverse:** AATCGGTTGGCTGTAGGA |  |  |
| **LEAP2B** | **Forward:** TGTGCCATTAGCGACTTCTGAG | 59.3 | KT625603 |
|  | **Reverse:** ATGATTCGCCACAAAGGGG |  |  |
| **β-defensin-1** | **Forward:** TTGCTTGTCCTTGCCGTCT | 58.4 | KT445868 |
|  | **Reverse:** AATCCTTTGCCACAGCCTAA |  |  |
| **MBL** | **Forward:** CAACTTCAATCAGATGTCAAACACC | 59.1 | KM275626 |
|  | **Reverse:** GCTCATATCTACAAAATGCCCTTCT |  |  |
| **TNFα** | **Forward:** CGCTGCTGTCTGCTTCAC | 58.4 | HQ696609 |
|  | **Reverse:** CCTGGTCCTGGTTCACTC |  |  |
| **IFNγ2** | **Forward:** TGTTTGATGACTTTGGGATG | 60.4 | JX657682 |
|  | **Reverse:** TCAGGACCCGCAGGAAGAC |  |  |
| **IL-1β** | **Forward:** AGAGTTTGGTGAAGAAGAGG | 57.1 | JQ692172 |
|  | **Reverse:** TTATTGTGGTTACGCTGGA |  |  |
| **IL-6** | **Forward:** CAGCAGAATGGGGGAGTTATC | 62.3 | KC535507 |
|  | **Reverse:** CTCGCAGAGTCTTGACATCCTT |  |  |
| **IL-8** | **Forward:** ATGAGTCTTAGAGGTCTGGGT | 60.3 | JN663841 |
|  | **Reverse:** ACAGTGAGGGCTAGGAGGG |  |  |
| **IL-10** | **Forward:** AATCCCTTTGATTTTGCC | 61.4 | HQ388294 |
|  | **Reverse:** GTGCCTTATCCTACAGTATGTG |  |  |
| **IL-11** | **Forward:** GGTTCAAGTCTCTTCCAGCGAT | 57.0 | KT445870 |
|  | **Reverse:** TGCGTGTTATTTTGTTCAGCCA |  |  |
| **IL-12p35** | **Forward:** TGGAAAAGGAGGGGAAGATG | 55.4 | KF944667 |
|  | **Reverse:** AGACGGACGCTGTGTGAGTGTA |  |  |
| **IL-12p40** | **Forward:** ACAAAGATGAAAAACTGGAGGC | 59.0 | KF944668 |
|  | **Reverse:** GTGTGTGGTTTAGGTAGGAGCC |  |  |
| **IL-15** | **Forward:** CCTTCCAACAATCTCGCTTC | 61.4 | KT445872 |
|  | **Reverse:** AACACATCTTCCAGTTCTCCTT |  |  |
| **IL-17D** | **Forward:** GTGTCCAGGAGAGCACCAAG | 62.3 | KF245426 |
|  | **Reverse:** GCGAGAGGCTGAGGAAGTTT |  |  |
| **IL-4/13A** | **Forward:** CTACTGCTCGCTTTCGCTGT | 55.9 | KT445871 |
|  | **Reverse:** CCCAGTTTTCAGTTCTCTCAGG |  |  |
| **IL-4/13B** | **Forward:** TGTGAACCAGACCCTACATAACC | 55.9 | KT625600 |
|  | **Reverse:** TTCAGGACCTTTGCTGCTTG |  |  |
| **TGF-β1** | **Forward:** TTGGGACTTGTGCTCTAT | 55.9 | EU099588 |
|  | **Reverse:** AGTTCTGCTGGGATGTTT |  |  |
| **TGF-β2** | **Forward:** TACATTGACAGCAAGGTGGTG | 55.9 | KM279716 |
|  | **Reverse:** TCTTGTTGGGGATGATGTAGTT |  |  |
| **NFκBp65** | **Forward:** GAAGAAGGATGTGGGAGATG | 62.3 | KJ526214 |
|  | **Reverse:** TGTTGTCGTAGATGGGCTGAG |  |  |
| **NFκBp52** | **Forward:** TCAGTGTAACGACAACGGGAT | 58.4 | KM279720 |
|  | **Reverse:** ATACTTCAGCCACACCTCTCTTAG |  |  |
| **c-Rel** | **Forward:** GCGTCTATGCTTCCAGATTTACC | 59.3 | KT445865 |
|  | **Reverse:** ACTGCCACTGTTCTTGTTCACC |  |  |
| **IκBα** | **Forward:** TCTTGCCATTATTCACGAGG | 62.3 | KJ125069 |
|  | **Reverse:** TGTTACCACAGTCATCCACCA |  |  |
| **IKKα** | **Forward:** GGCTACGCCAAAGACCTG | 60.3 | KM279718 |
|  | **Reverse:** CGGACCTCGCCATTCATA |  |  |
| **IKKβ** | **Forward:** GTGGCGGTGGATTATTGG | 60.3 | KP125491 |
|  | **Reverse:** GCACGGGTTGCCAGTTTG |  |  |
| **IKKγ** | **Forward:** AGAGGCTCGTCATAGTGG | 58.4 | KM079079 |
|  | **Reverse:** CTGTGATTGGCTTGCTTT |  |  |
| **TOR** | **Forward:** TCCCACTTTCCACCAACT | 61.4 | JX854449 |
|  | **Reverse:** ACACCTCCACCTTCTCCA |  |  |
| **S6K1** | **Forward:** TGGAGGAGGTAATGGACG | 54.0 | EF373673 |
|  | **Reverse:** ACATAAAGCAGCCTGACG |  |  |
| **4EBP1** | **Forward:** GCTGGCTGAGTTTGTGGTTG | 60.3 | KT757305 |
|  | **Reverse:** CGAGTCGTGCTAAAAAGGGTC |  |  |
| **4EBP2** | **Forward:** CACTTTATTCTCCACCACCCC | 60.3 | KT757306 |
|  | **Reverse:** TTCATTGAGGATGTTCTTGCC |  |  |
| **TLR1** | **Forward:** AAGAACGCAGACTGGGTCAAAG | 59.4 | FJ542041 |
|  | **Reverse:** TGCTATCCATCCCTCGCATTAC |  |  |
| **TLR2** | **Forward:** GGGCTCACTCATCTGGACG | 59.8 | FJ542042 |
|  | **Reverse:** CCTTCTTTCGCCGCTTTG |  |  |
| **TLR4** | **Forward:** GCTGCCCATTATTCCTTCATC | 58.9 | FJ542043 |
|  | **Reverse:** TCCACCTATTCATCTTTGCCTTC |  |  |
| **TLR5** | **Forward:** TGACGCAGCAAATGTTCAAGC | 58.7 | KF736231 |
|  | **Reverse:** GAGAACCTGGGAGCAAAGCAA |  |  |
| **MyD88** | **Forward:** CAAATGATGGACTTTACCTACCTG | 59.5 | FJ843088 |
|  | **Reverse:** ACATCTTTCCTTTCGGCTTT |  |  |
| **TRIF** | **Forward:** ATACCCGTTTGTTGTCTCAG | 57.8 | KC333648 |
|  | **Reverse:** TGGATTCTGTAGCAGTTGGC |  |  |
| **TRAF6** | **Forward:** TCACTCACTGTCAGATGTC | 56.9 | KC465198 |
|  | **Reverse:** TGTTGGCTCTTGTGTTCA |  |  |
| **IRAK1** | **Forward:** ATACAGGCATCCCAACATAATGG | 59.6 | JQ239168 |
|  | **Reverse:** ACAGCGTAGTCGGTCTTCTAAAG |  |  |
| **IRAK4** | **Forward:** CTCCACACTGAGAGCTTTATC | 58.7 | MK519390 |
|  | **Reverse:** ATGTGCAGCTGTGTGTATCT |  |  |
| **β-actin** | **Forward:** GGCTGTGCTGTCCCTGTA | 61.4 | M25013 |
|  | **Reverse:** GGGCATAACCCTCGTAGAT |  |  |
| **GAPDH** | **Forward:** GTTACAAGGGAGAAGTTCACCAT | 58.0 | GQ266395 |
|  | **Reverse:** CCGGTAGACTCGACTACATACAG |  |  |

^1^ LEAP-2, liver expressed antibacterial peptide 2; MBL, mannose-binding lectin; TNF-α, tumor necrosis factor α; IFNγ2, interferon γ2; IL, interleukin; TGFβ, transforming growth factor β; NFκB p65, nuclear factor kappa B p65; IκBα, inhibitor of κBα; IKK, IκB kinase; TOR, target of rapamycin; S6K1, ribosomal protein S6 kinase 1; 4EBP, eIF4E-binding protein; TLRs, Toll like receptors; MyD88, myeloid differentiation primary response 88; TRIF, toll-like receptor (Tlr) 4 adaptor TIR-domain-containing adapter-inducing interferon-β; TRAF6, TNF receptor associated factor 6; IRAK1, interleukin-1 receptor-associated kinase 1.

**Supplementary Table 4. The information of antibodies (Immunohistochemical)**

| Indices | Host | Source | Catalog No. | Dilution for WB |
| --- | --- | --- | --- | --- |
| p-IRAK1 | Rabbit | Affinity (Golden, Colorado, USA) | AF4443 | 1:150 |
| MyD88 | Rabbit | ABclonal (Wuhan, China) | A0786 | 1:200 |
| TRAF6 | Rabbit | ABclonal (Wuhan, China) | A0973 | 1:150 |
| NF-κB p65 | Rabbit | Affinity (Golden, Colorado, USA) | AF5006 | 1:200 |

**Supplementary Table 5. The information of antibodies (Western blot)**

| Indices | Host | Source | Catalog No. | Dilution for WB |
| --- | --- | --- | --- | --- |
| TLR2 | Rabbit | ABclonal (Wuhan, China) | A11225 | 1:900 |
| p-IRAK1 | Rabbit | Affinity (Golden, Colorado, USA) | AF4443 | 1:1000 |
| MyD88 | Rabbit | ABclonal (Wuhan, China) | A0786 | 1:1000 |
| TRAF6 | Rabbit | ABclonal (Wuhan, China) | A0973 | 1:1000 |
| β-actin | Rabbit | Affinity (Golden, Colorado, USA) | AF7018 | 1:3000 |
| LaminB1 | Rabbit | Affinity (Golden, Colorado, USA) | AF5161 | 1:1000 |
| Total TOR | Rabbit | Affinity (Golden, Colorado, USA) | AF6308 | 1:1000 |
| p-TOR Ser 2448 | Rabbit | Affinity (Golden, Colorado, USA) | AF3308 | 1:1000 |
| NF-κB p65 | Rabbit | Affinity (Golden, Colorado, USA) | AF5006 | 1:750 |
